# Atopy in chronic urticaria: an important yet overlooked issue

**DOI:** 10.3389/fimmu.2024.1279976

**Published:** 2024-02-06

**Authors:** Qiquan Chen, Xianjie Yang, Bing Ni, Zhiqiang Song

**Affiliations:** ^1^ Department of Dermatology, Southwest Hospital, Army Medical University, Chongqing, China; ^2^ School of Medicine, Chongqing University, Chongqing, China; ^3^ Department of Pathophysiology, Army Medical University, Chongqing, China

**Keywords:** chronic urticaria, chronic spontaneous urticaria, chronic inducible urticaria, atopy, IgE, mast cell

## Abstract

Chronic urticaria (CU) is one of the most common dermatological diseases and has a significant impact on the quality of life of patients. However, the pathogenesis of this disease remains unclear. Autoimmunity in chronic spontaneous urticaria (CSU) has received considerable attention and has been studied previously. Atopy is an important characteristic of CU; however, it has not been fully recognized. Atopy predisposes individuals to immune responses to allergens, leading to type 2 inflammation and immunoglobulin E (IgE) overproduction. Compared with healthy individuals, patients with CU have a higher proportion of atopy, and an atopic background is correlated with the clinical characteristics of CU. The total IgE levels in patients with CU is significantly higher than those in healthy individuals. Although its level is not higher than that in classic allergic diseases, it is closely related to CU. Exogenous allergens, auto-allergens, and specific IgEs, which are closely related to atopy, have been reported, and their roles in CU pathogenesis are also being studied. Local and systemic atopic inflammation is present in patients with CU. This review summarizes the current knowledge regarding atopy and CU, speculating that there are CU subtypes, such as atopic CSU or atopic chronic inducible urticaria (CIndU) and that atopy may be involved in the pathogenesis of CU. These findings provide a new perspective for a comprehensive understanding of the clinical features of CU and further research regarding its pathogenesis.

## 1 Introduction

Chronic urticaria (CU) is a pruritic skin disease defined as the occurrence of evanescent wheals, angioedema, or both for more than six weeks. It can be divided into chronic spontaneous urticaria (CSU) and chronic inducible urticaria (CIndU) (1, 2). Due to its long course and recurrent symptoms, CU imposes a great burden on patients, patient families, healthcare systems, and society as a whole (3). The pathogenesis of CU is not fully understood. Since the 1990s, several studies have focused on autoimmunity in CSU, identifying a potential autoimmune etiology in up to 50% of patients with this condition (4). However, the autoimmune theory does not fully explain the pathogenesis of CSU and is less relevant for CIndU. A large proportion of CU cases are of unknown etiology, and studies regarding the etiology and mechanism of CIndU are rare. Atopy is a predisposition to immunological responses to allergens, leading to type 2 inflammation and immunoglobulin E (IgE) overproduction (5). Patients with atopy often present with allergen-specific IgE, elevated total IgE levels, or a definite history of atopic diseases (6). Compared to other typical allergic diseases (such as atopic dermatitis (AD), allergic conjunctivitis, allergic rhinitis (AR), and asthma), atopy is often considered less important in the setting of CU. However, increasing evidence has shown that atopy is a predisposing factor for CU. Targeting free IgE and its receptor with antibodies provides an effective treatment for CSU and most subtypes of CIndU (7), resulting in a strong association between atopy and CU. This review focuses on the research progress of atopy in the clinical aspects, etiology, and pathophysiology of CU.

## 2 High prevalence of atopy in CU

Individuals with atopy frequently have one or more atopic disorders, such as AR, asthma, AD, and allergic conjunctivitis. Patients with CU commonly present with atopic diseases. Nassif (8) reported that 82 of 85 patients with CU had a personal and/or familial atopic status (asthma, eczema, or AR/allergic conjunctivitis history), suggesting that CU may be part of the atopic diathesis, in addition to asthma, eczema, and AR/allergic conjunctivitis. To further reveal the relationship between atopic disorders and CU, Shalom et al. (9) conducted a large cross-sectional study of 11,271 patients with CU and 67,216 controls, revealing that 19.9%, 10.8%, and 9.8% of patients with CU had AR, asthma, and AD, respectively, compared to 10.1%, 6.5%, and 3.7% of controls (all significantly different). Another population-based retrospective cohort study from Taiwan included 9,332 patients with CU and 37,328 controls, and reported that CU was significantly associated with AD and AR (10). Similarly, a study of 1,108,833 adolescents from Israel found that individuals with CSU were significantly more likely to have allergic diseases, including food allergy (odds ratio (OR): 7.31, 95% confidence interval (CI): 6.13-8.72), AR (OR: 2.9, 95% CI: 2.71-3.11), AD (OR: 2.35, 95% CI: 2.03-2.72), and asthma (OR: 1.46, 95% CI: 1.35-1.57) (11). Kitsioulis et al. reported that children with a history of an early diagnosis of AD were at an increased risk of later CSU occurrence (OR: 2.923, 95% CI: 1.647-5.189, p < 0.001) (12). Taken together, these studies suggest a significant association between CU and atopic diseases.

In addition to the concomitant presence of atopic diseases, sensitization to allergen-specific IgE, which is an important indicator of atopy, is also a concern in patients with CU. The positive sensitization rate of patients with CU to various allergens according to the skin prick test (SPT) or serum-specific IgE test ranged from 17.2–95.83% (**Table 1**), which was significantly higher than that of healthy individuals (13–33). Among the allergens identified in patients with CU, house dust mites (HDM) are often the most common (13, 14, 16, 26, 29), followed by weed (mugwort and ragweed) and tree pollen, cereal pollen, cockroaches, weeds, cat hair, rat, and *Candida albicans* and mixed molds (17, 19, 21, 22, 31, 32). In addition, an association between CU and Anisakis simplex (As) sensitization has been reported, and the sensitization rates to pollen, dander, and mold in patients with CU with sensitization to As (CU/As+) were significantly higher than those in patients without As sensitization (CU/As-) (18). Multiple allergen sensitization is more common than single allergen sensitization in patients with CU, and sensitization to one allergen in patients with CU may lead to subsequent sensitization to other allergens (18, 34).

**Table 1:** Studies exploring the allergen sensitization in CU.

Author (year)	Type of CU	No. of patients	Categories of allergens and positive rate	Detection method	Total presence of atopy	History of atopy	Clinic correlation
Caliskaner et al (13)	CUA	259	Mites (24.7%), Pollens (7.7%), Molds (0.4%), Animal dander (0), Cockroach (0.8%)	SPT	27.4%	Patients with a history of atopy were excluded	Not given
Mahesh et al (14)	CU	122	HDM	SPT	64%	Patients with a history of allergic rhinitis (28.7%), asthma (23.8%), contact dermatitis (2.5%), drug allergies (3.3%), conjunctivitis (0.8%)	Not given
Kulthanan et al (15)	COU	88	16 food allergens and 12 aeroallergens	SPT	47.7%	Personal history of atopy (31.8%), family history of atopy (33%)	Not given
Kulthanan et al (16)	CIU	172	HDM	SPT	34.9%	Personal history of atopy (28.5%), family history of atopy (26.7%)	Little clinical relevance (3.3%) to their urticarial symptoms.
Staubach et al (17)	CSU	112	Candida albicans (CA) and mold allergens	Intracutaneous skin testing (IST); specific IgE	IST: 45% specific IgE: 13%	Not given	Not given
Daschner et al (18)	CU	129	Aeroallergens	SPT	CU/ As+:60.4% CU/ As-: 45%	21.3% of CU/As- and 42.2% of CU/As+ suffered from RCBA	Not given
Refaat et al (19)	CIU	174	Common aeroallergens mites (13.8%), pollens (5.2%), mixed molds (4%)	SPT and specific IgE	17.2%	Respiratory allergic diseases were excluded	Not given
Augey et al (20)	CU	128	Aeroallergens and food allergens	SPT and specific IgE	46.7%	Personal, familial (first-degree relatives), familial and/or personal histories of atopic diseases were reported by 42%, 55.5%, 68% of the CU patients	Not given
de Vos et al (21)	CU	37	Mugwort pollen	SPT	18.9%	CU patients with coexisting allergic rhinitis were more likely to be sensitized to mugwort as subjects not suffering from CU (67 vs. 30%; p = 0.004).	Not given
Gecer et al (22)	CIU	50	Aeroallergens, HDM (16%), mixture of 4 cereals (16%), mixture of 5 grasses (12%), mixture of 12 grasses (12%)	SPT	32%	Not given	Not given
Song et al (23)	CSU	862	HDM	SPT	17.7%	Not given	Patients with positive SPT had higher UAS and DLQI values
Bains et al (24)	CIU	41	130 allergens (66 aeroallergens and 64 food allergens), dust and pollen (26.83%), food (21.9%), insects (17.07%), fungus (12.20%), Dermatophagoides farinae (7.32%)	SPT	63.41%	Personal and family history of atopy was reported in 14.63 and 9.76%, respectively	Not given
Altrichter et al (25)	CholU	30	Inhaled allergens	Specific IgE	57%	Not given	Atopy was linked to high CholU severity, activity and impact on quality of life
Lee et al (26)	AU and CU	218	Common aeroallergens	Specific IgE	54%	Not given	CU have a higher prevalence of sensitization to HDM and polysensitization than acute urticaria
Altrichter et al (27)	CSU	49	Staphylococcus aureus enterotoxins (SEs), SEB	Specific IgE	51%	Not given	SEB-IgE levels were strongly correlated with disease activity in CSU patients, BHR in response to SEB was clinically correlated with duration of disease
Latif et al (28)	CSU	70	Aeroallergens and food allergens, HDM is the mostly frequent allergen (34.8%)	Specific IgE	65.7%	Patients were without other allergic diseases	Not given
Zhou et al (29)	pediatric patients with CU	96	34 aeroallergens and food allergens, dust mites and insects (16.67%), main allergens (10.42%), food additives (8.33%)	Specific IgE	95.83%	Not given	Not given
Park et al (30)	CU in children	253	Dermatophagoides pteronyssinus, D. farinae, egg white, cow’s milk, wheat, soybean, and peanut	SPT and specific IgE	41.1%	Personal history of atopy (≥20.1%), family history of atopy (≥15.0%)	Allergic sensitization was a risk factor associated with longer duration CU
Ping et al (31)	CU	818	Inhaled and food allergens, D. pteronyssinus (34.5%), cockroach (12.5%) and tree pollen mix (11.1%)	Specific IgE	48.0%	Not given	Positive rates for the majority of allergens were higher in males than in females and were significantly different between age groups
Esmaeilzadeh et al (32)	CSU	91	The most prevalent aeroallergens	SPT	82.4%	40.7% of the patients had allergic rhinitis	Not given
Chen et al (33)	CIndU	168	34 aeroallergens and food allergens	SPT	42.9%	16.7% of CIndU patients had a personal history of atopy	The allergen sensitization and atopic disease history of CIndU patients were associated with disease severity, pruritus intensity, quality of life, and the proportion of cases with complicated angioedema

CU, chronic urticaria; CUA, chronic urticaria and angioedema; CIU, chronic idiopathic urticaria; CSU, chronic spontaneous urticaria; AU, acute urticaria; CIndU, chronic inducible urticaria; CSPT, skin prick test; HDM, house dust mite; COU, chronic ordinary urticaria, excluding physical urticaria and other special types of urticaria; As, sensitization against Anisakis simplex; RCBA, allergic rhinoconjunctivitis and/ or bronchial asthma; CholU, cholinergic urticaria; UAS, urticaria activity score; DLQI, Dermatology Life Quality Index; SEB, staphylococcal enterotoxin B; BHR, basophil histamine release.

The rates of allergen sensitization were significantly higher in patients with CU, regardless of the presence or absence of a history of atopy (14, 21). Among patients with CU without comorbid atopic disorders, the proportion of patients sensitized to exogenous allergens is high. Caliskaner et al. (13) reported that 27.4% of patients with CU without AR and/or asthma are sensitized to one or more inhaled allergens, which is significantly higher than that in healthy controls (7%). In addition, there are differences in the prevalence of atopy among the different CU subtypes. Cholinergic urticaria (CholU) and cold urticaria have higher rates of atopic predisposition, with the highest reported rates of 57% and 89.3%, respectively (25, 35, 36). In a recent atopy analysis of CIndU subtypes, including CholU, symptomatic dermatographism, cold contact urticaria, and heat contact urticaria, no significant differences in the SPT-positive rates were observed between these four subtypes, though CholU had the highest rate of atopic history (57.1%) (33). No comparative studies regarding the proportions of CholU and CSU atopy have been reported, and the prevalence of atopy in other CU subtypes requires further investigation.

## 3 Clinical relevance of allergen sensitization to CU

Sensitization to allergens is a hallmark of atopy; however, its clinical relevance to CU has not been adequately studied, and the existing findings are controversial. As previous studies have found that positive allergens are not typically associated with CU symptoms and avoidance of these allergens does not prevent recurrence, CU is not considered an allergic disease (20).

However, this may not be accurate. Recurrent urticaria associated with dietary allergies is frequently diagnosed as CU, and previous studies have shown that IgE sensitization via wheat and barley allergens is a causative factor for CU (37, 38). Allergen immunotherapy, a treatment that induces tolerance to specific allergens, has been reported to relieve urticaria and respiratory symptoms in patients with concomitant CU and respiratory allergies (39, 40). In contrast, the exacerbation of urticaria symptoms during HDM immunotherapy for CU has been reported (41). Acetylsalicylic acid sensitization has been reported to cause CU via desensitization (42). These reports suggest that allergens may be causative or deteriorating factors in patients with CU.

Increasing evidence suggests that allergen sensitization is clinically relevant to CU. As summarized in **Table 1**, Kulthanan et al. reported clinical correlations between allergen sensitization and urticaria symptoms (16), and Song et al. (23) reported more conclusive evidence. This study investigated the correlation between HDM IgE sensitization and clinical status in 862 patients with CSU and reported that the disease activity of patients with HDM sensitization was significantly higher than that of non-sensitized patients. Furthermore, 23 patients with strong sensitization to HDM had significantly aggravated urticaria symptoms when they continued to live in a room with high HDM density, while their symptoms significantly improved when the HDM exposure was avoided (23). Altrichter et al. (27) reported that levels of staphylococcal enterotoxin B (SEB)-specific IgE were strongly associated with disease activity in patients with CSU, while the degree of basophil histamine release (BHR) induced by SEB was clinically correlated with disease duration. In addition, a retrospective cohort study including 4,552 patients with CU reported that HDM sensitization is an important factor affecting the remission of CU (43). Another Korean study confirmed that children sensitized to common inhaled and food allergens had a longer natural course of CU (30), indicating that allergen sensitization may be related to the poor prognosis of CU. In a recent study regarding atopy and CIndU, patients with CIndU and allergen sensitization or a history of atopic disease had a longer natural history than those non-atopic individuals, though the difference was not significant (33).

The distribution of allergen sensitization in patients with urticaria appears to be related to sex. Kulthanan et al. (16) reported that HDM sensitization was more common in male patients with CU than in female patients, and Ping et al. demonstrated the same sex distribution difference in atopy in patients with CU in a larger sample study (31). CU and acute urticaria had different allergen sensitization spectra; however, the reason for this phenomenon remains unclear (31). Some CU subtypes are more closely associated with atopy. Patients with an atopic predisposition had higher CholU severity, activity, and impact on quality of life, as well as different comorbidity profiles and seasonal exacerbation patterns compared to patients without an atopic predisposition (25). In addition, sweat allergy, a type I hypersensitivity reaction to sweat components, is observed in patients with CholU and AD (44). Sweat allergy is closely related to the clinical manifestations and treatment response of CholU and is a key factor in the CholU classification (45).

## 4 High total IgE and its clinical relevance in CU

Atopy is the familial or individual tendency to overproduce IgE antibodies. Therefore, elevated serum total IgE levels are generally considered important markers of atopy (46). Elevated total serum IgE levels in patients with CU were first reported by Greaves et al. approximately 50 years ago (47). Subsequently, an increasing number of studies have confirmed this phenomenon (48). A high proportion (18-82%) of patients with CSU have elevated serum total IgE levels, though these levels were lower than those in patients with classic allergic diseases, such as AD (48–50). The IgE level of patients with CSU and AD are significantly higher than those of patients with CSU without AD (51). In addition, another study reported that the expression of IgE high-affinity receptors (FcϵRI) on basophils of patients with CIndU, regardless of the subtype, is significantly increased and correlated with the response to omalizumab treatment (52). Although it was not observed that the total serum IgE level was significantly higher in patients with CIndU than in healthy individuals, the total serum IgE level was higher in patients resistant to antihistamines (52). More studies regarding the total serum IgE levels in patients with CIndU and its subtypes are needed.

The total IgE levels vary considerably among different types of CSU. Autoimmunity plays an important role in the pathogenesis of CSU, which is divided into autoimmune CSU (aiCSU) and non-autoimmune CSU. aiCSU can be further divided into type I (also known as auto-allergic CSU) and type IIb aiCSU (53). The presence of auto-IgE is a hallmark of auto-allergic CSU, and studies have shown that the total IgE level and the proportion of atopy in patients with CSU and auto-IgE are significantly higher than those patients with CSU without auto-IgE (54–56). In contrast, patients with type IIb aiCSU typically have very low levels of total IgE and often have a positive autologous serum skin test (ASST) or positive thyroid autoantibodies (57). Some patients have positive ASST and SPT, though the total IgE level in these patients was lower than that in patients with negative ASST and positive SPT (23).

The correlation between the total IgE level and the disease characteristics of patients with CSU is unclear. Elevated serum total IgE levels in patients with CSU are positively correlated with disease severity and the natural course of the disease (49). Choi et al. reported that total IgE levels are significantly and positively correlated with the Urticaria Activity Score (UAS) and Chronic Urticaria Quality of Life Questionnaire score (58). However, a cluster study reported no significant correlation between serum total IgE levels and CU remission or relapse (43). This association may differ in different patient populations. Kocaturk et al. reported that symptoms were more severe in pregnant patients with CSU with low total IgE levels (59). Although patients with type IIb aiCSU usually have low total IgE levels, they are commonly characterized by a high degree of disease severity (60). Therefore, the relationship between total IgE levels and the severity of CSU disease is controversial and only consistent with specific circumstances.

The relationship between total serum IgE levels and treatment response in patients with CSU is of great interest. Previous studies have not found a correlation between serum total IgE levels and response to antihistamines in patients with CSU (51, 61). However, patients with CIndU with higher total IgE levels are resistant to antihistamine treatment (52). The total IgE levels are associated with cyclosporine treatment outcomes, as patients with high total IgE levels have a poor response to cyclosporine treatment (62). The relationship between total IgE levels and omalizumab treatment has been widely reported, and includes treatment response (63), onset time (64), and relapse after drug withdrawal (65). Overall, the total IgE level can be used as a reference for administering omalizumab or cyclosporine for the treatment of refractory CSU as a high level of total IgE predicts that the patient is likely to respond well to omalizumab and be resistant to cyclosporine.

Free IgE cannot be distinguished from complex IgE in the circulation using conventional methods (66). Serum-free IgE levels are more predictive of atopic status than total IgE levels (46). Jang et al. (67) recently developed a novel assay to measure serum-free IgE levels in patients with CSU. Patients with CSU with atopy had significantly higher serum free IgE levels than those without atopy (67), while no associations were noted with UAS, urticaria duration, or response to omalizumab. However, patients with elevated levels of Der p-specific IgE and its ratio to the level of serum total free IgE have favorable responses to omalizumab (67). Therefore, the clinical relationship between serum-free IgE levels and CSU requires further confirmation.

## 5 Exogenous allergens and auto-allergens in CU

Allergen sensitization is one of the most prominent signs of atopy. Similar to classic type I hypersensitivity involving exogenous allergens, mechanisms of mast cell (MC) activation and degranulation may also be present in the setting of CU. Specific targets of IgE-induced MC degranulation in patients with CU may be exogenous or endogenous.

IgE targeting classic exogenous allergens, such as aeroallergens and food allergens, has been detected in some patients with CSU; however, its pathogenic function remains unclear and controversial (15, 21, 68). Most exogenous allergen exposures do not directly induce CU symptoms, and specific IgE levels are not believed to be related to CU symptoms. However, Altrichter et al. reported that specific IgE against SEB are common and functional in patients with CSU, as determined using the BHR test (27). However, the proportion of histamine release induced by SEB-specific IgE among the total amount of histamine released in patients with CSU is very low and not enough to induce the occurrence and recurrence of CSU symptoms, suggesting that either the occurrence of CSU requires the simultaneous stimulation of multiple exogenous allergen-specific IgEs or that these specific IgEs trigger inflammation by acting on inflammatory cells other than MCs (69, 70). In addition, more than 50% of the patients with CU tested positive for nasal *Staphylococcus aureus*, which may be the underlying cause of SEB sensitization (71, 72). Overall, more research regarding the involvement of exogenous allergens in the pathogenesis of CU is necessary.

Auto-allergens, also known as endogenous allergens, have attracted increasing attention in research regarding the pathogenesis of CU. Bar-Sela et al. detected IgE autoantibodies against thyroid peroxidase (TPO) in patients with CU (73), and several other studies have detected IgE-type antithyroid autoantibodies (AAbs), including anti-TPO IgE and anti-thyroglobulin IgE, in different populations and races of patients with CSU (55, 56, 74–79). In 2019, Sanchez et al. confirmed the pathogenic role of anti-TPO IgE in patients with CSU using *in vitro* and *in vivo* tests (55). Sanchez et al. reported that peripheral blood basophil CD203c expression was significantly increased in patients with anti-TPO IgE-positive CSU after exposure to TPO as well as significant positive responses in intradermal and SPT with TPO (55). Urticarial wheals are also induced by transferring serum containing anti-TPO IgE to healthy controls (55). A study of patients with urticaria intolerant to aspirin reported similar results (56). In addition to thyroid-related autoantigens and IgE, more autoantigens and specific IgEs associated with CU have been identified (**Table 2**). Eosinophil cationic protein and eosinophil peroxidase (EPX)-specific IgE antibodies have been observed in patients with severe CSU (77). The extracellular domain of TPO shares approximately 45% similarity with the myeloperoxidase of eosinophils (84). An IgE cross-reaction between EPX and TPO and IgE sensitization to EPX preceded sensitization to TPO (77). Kashiwakura et al. reported that the anti-dsDNA IgE levels in patients with CU were significantly higher than those in healthy individuals, and its role in the pathogenesis of CU was confirmed by the basophil activation test (80). Cugno et al. first reported that tissue factor (TF)-specific IgE antibodies were elevated in patients with CSU and that these antibodies could functionally mediate the release of leukotriene C4 (LTC4) from peripheral blood basophils upon TF stimulation (79). Asero et al. detected the levels of IgE autoantibodies to FcϵRI exceeding the upper normal limit in six patients (30%) via sandwich enzyme-linked immunosorbent assay. Therefore, FcϵRI may be a novel auto-allergen in patients with CSU (81). Most recently, Su et al. reported that one in five patients with CSU has serum IgE specific for tissue transglutaminase 2 (TG2), an autoantigen. Functional assays were used to confirm that TG2- and TG2-specific IgE can induce the activation and degranulation of human skin MCs (82). In contrast to other studies, Schmetzer et al. detected over 200 autoantigens recognized by IgE in the serum of patients with CSU using microarray analyses, of which eight autoantigens were accessible in the skin, including IL-24. Among the autoantigen-specific IgE antibodies, IL-24 has the highest level of specific IgE in the serum (83) and can induce the degranulation of sensitized MCs from the serum of patients with CSU, but not in MCs incubated with serum from healthy control patients (83). As an increasing number of autoantigens and corresponding IgEs have been identified, their clinical value in CU has attracted attention. Although anti-TPO IgE is not a specific biomarker for CSU, it plays a pathogenic role in inducing effector cell activation and skin exacerbation in some patients with CSU (55). Elevated IgE anti-FcϵRI in patients with CSU may be associated with late-and non-responses to omalizumab treatment (81, 85), which serve as predictors of response to omalizumab. Anti–IL-24 IgE levels are associated with disease activity (83). However, other studies have reported no clinical relevance of autoantigens or the corresponding IgE levels. Therefore, additional studies are needed to identify the clinical value of autoantigens and their corresponding IgEs in patients with CU.

**Table 2:** Studies exploring autoallergens in CU.

Author (year)	Autoallergen	No. of patients / Type of CU	Atopy of the patients	Type of assay	Cut-off (IU/mL)	Positive rate	IgE-anti-autoallergen levels (median or mean: IU/mL)	Control group	Clinic correlation
Bar-Sela et al (73)	TPO	6 / CU	Total IgE 450 IU/mL	ELISA	ND	1/6	>500 IU/mL	None	The association between CU and thyroid autoimmunity in patients with high titers of specific IgE autoantibodies should be further investigated.
Tedeschi et al (74)	TPO	35 / CU	ND	Radioimmunoassay	ND	0	ND	None	The detection of IgE specific for TPO is an occasional finding and that these antibodies are unlikely to play a pathogenic role in most cases of CU.
Concha et al (75)	TPO / TG	20 / CU	ND	ELISA	ND	2/20	ND	None	IgE anti-thyroid antibodies do not appear to play a causal role in urticaria in the majority of patients
Altrichter et al (76)	TPO	478 / CSU	ND	ELISA	5.0 IU/ml	54.2%	Median 5.50, IQR 3.2–7.7)	Median 1.46, IQR 0.27–4.45	A sizeable subgroup of CSU patients expresses IgE antibodies against TPO.
Shin et al (56)	TPO	96 / AICU	50% atopy	ELISA	0.261 (O.D. value)	7.5%	Value: 0.157±0.344 (O.D. value)	Positive rate: 0% value: 0.111±0.050 (O.D. value)	It is suggested that specific IgE to TPO play a pathogenic role in AICU.
Sánchez et al(55)	TPO	100 / CSU	42% atopy	Flouroenzyme immunoassay	The mean and 3 standard deviations of absorbance values from 40 healthy controls	34%	ND	ND	Anti-TPO IgE is not a specific biomarker for CSU, but it plays a pathogenic role in inducing effector cell activation and skin exacerbation in some patients with CSU.
Sánchez et al (77)	TPO	56 / CSU	56.3% atopy	ELISA	The mean and 3 standard deviations of absorbance values from 60 healthy controls	27.2%	ND	ND	Explain thyroid problems in CSU patients.
Zhang et al (78)	TPO / TG	1100 / CSU	Patients with active atopic diseases were excluded	immunoblotting	0.7 kU/L	TPO: 18.0% TG: 7.0%	ND	TPO: 6.8% TG: 2.9%	Highlights the correlation between IgE and IgG anti-thyroid AAbs.
Sánchez et al (77)	EPX / ECP	56 / CSU	56.3% atopy	ELISA	The mean and 3 standard deviations of absorbance values from 40 healthy controls	EPX: 10.9% ECP: 5.4%	ND	EPX: 3.6% ECP: 1.6%	In the CSU group, there was a correlation between the anti-EPX IgE and anti-TPO IgE levels
Hatada et al (80)	dsDNA	85 / CU	Total IgE 307±318 IU/ml	ELISA	ND	anti-dsDNA IgE level in CU was significantly increased	ND	Significant lower than that in CU	dsDNA can activate of basophils from patients with CU who tested positive for anti-dsDNA IgE
Cugno et al (79)	TF / TG	15 / CSU	8/15 total IgE >100 KU/L	ELISA	TF: 475 OD TG: 278 OD	TF: 60% TG: 87%	TF: 688 [415-815] OD TG: 520 [331-815] OD	TF: 326 [129-424] OD TG: 184 [170-220] OD	Elevated IgE to TF and TG are abated by omalizumab
Asero et al (81)	FcϵRI	20 / CSU	ND	ELISA	290 OD	30%	22–839 OD	4–290 OD	Late responders to omalizumab showed higher levels of IgE anti-FcϵRI than early responders
Su et al (82)	TG2	160 / CSU	ND	ELISA	77.7 AU	20.6%	Median: 10.8 AU, IQR 5.0-58.5 AU,	Median: 6.7 AU, IQR 1.3-28.7 AU	Elevated IgE-anti-TG2 levels were not linked to any demographic, clinical and laboratory features of CSU including the presence of IgE-anti-TPO and IgE-anti-IL-24
Schmetzer et al (83)	IL-24	1062 / CSU	ND	ELISA	0.33 IU/mL	80%	0.52 ± 0.24 IU/mL	0.27 ± 0.08 IU/mL	IgE-anti–IL-24 levels showed an association with disease activity, as assessed by the urticaria activity score and with reduced basophil counts.

TPO, thyroid peroxidase; ELISA, enzyme-linked immunosorbent assay; ND, not described; TG, thyroglobulin; IQR, interquartile range; AICU, aspirin intolerant chronic urticaria; AAbs, anti-thyroid autoantibodies; EPX, eosinophil peroxidase; ECP, eosinophil cationic protein; TF, tissue factor; FcϵRI, high affinity IgE receptor; TG2, tissue transglutaminase 2; AU, arbitrary units; OD, optical density.

Few studies regarding the roles of exogenous or endogenous allergens in patients with CIndU have been reported. Hide et al. initially reported that more than half of the basophils from patients with CholU had increased histamine release when stimulated with semi-purified sweat antigens, though basophils from healthy individuals did not (86). They further found that the serum levels of specific IgE against MGL_1304, the major allergen in human sweat, were significantly higher in patients with CholU than in healthy controls (87). MGL_1304 is secreted by *Malassezia globosa* and induces the release of histamine from human basophils (88). Auto-allergy is considered an important mechanism of CIndU. In some patients, such as those with solar urticaria, relevant autoantigens have been identified and characterized using IgE (89). In addition, some factors of CIndU, including those associated with cold contact urticaria, symptomatic dermographism, and solar urticaria, are transferable, and the transferable factors in the serum may be specific IgE against a currently unknown auto-allergen (90–92). In CIndU, an appropriate trigger may lead to the production of auto-allergens that increase specific IgE levels, resulting in MC degranulation (93).

## 6 IgE in the pathogenesis of CU

The pathogenesis of CU remains unclear. IgE involved in atopy in patients with CU includes endogenous and exogenous allergen-specific IgE and elevated total IgE (or free IgE) levels. Exogenous allergens, auto-allergens, and their specific IgEs are mainly involved in the pathogenesis of CU through classic type I hypersensitivity (94). IgE is traditionally believed to induce MC sensitization rather than activation. However, basophil CD63 expression was significantly elevated in patients with CSU sensitized to allergen-specific IgE, even in the absence of allergen exposure (95). In addition, several studies conducted in the past twenty years have reported that high concentrations of IgE or sensitization with IgE alone may induce a variety of reactions without allergens in human basophils, human MCs, human cell lines, bone marrow-derived MCs, and rat basophilic leukemia cells (RBL-2H3) (96–101). These findings provide new insights regarding the mechanism underlying atopy observed in patients with CU.

**Figure 1:**
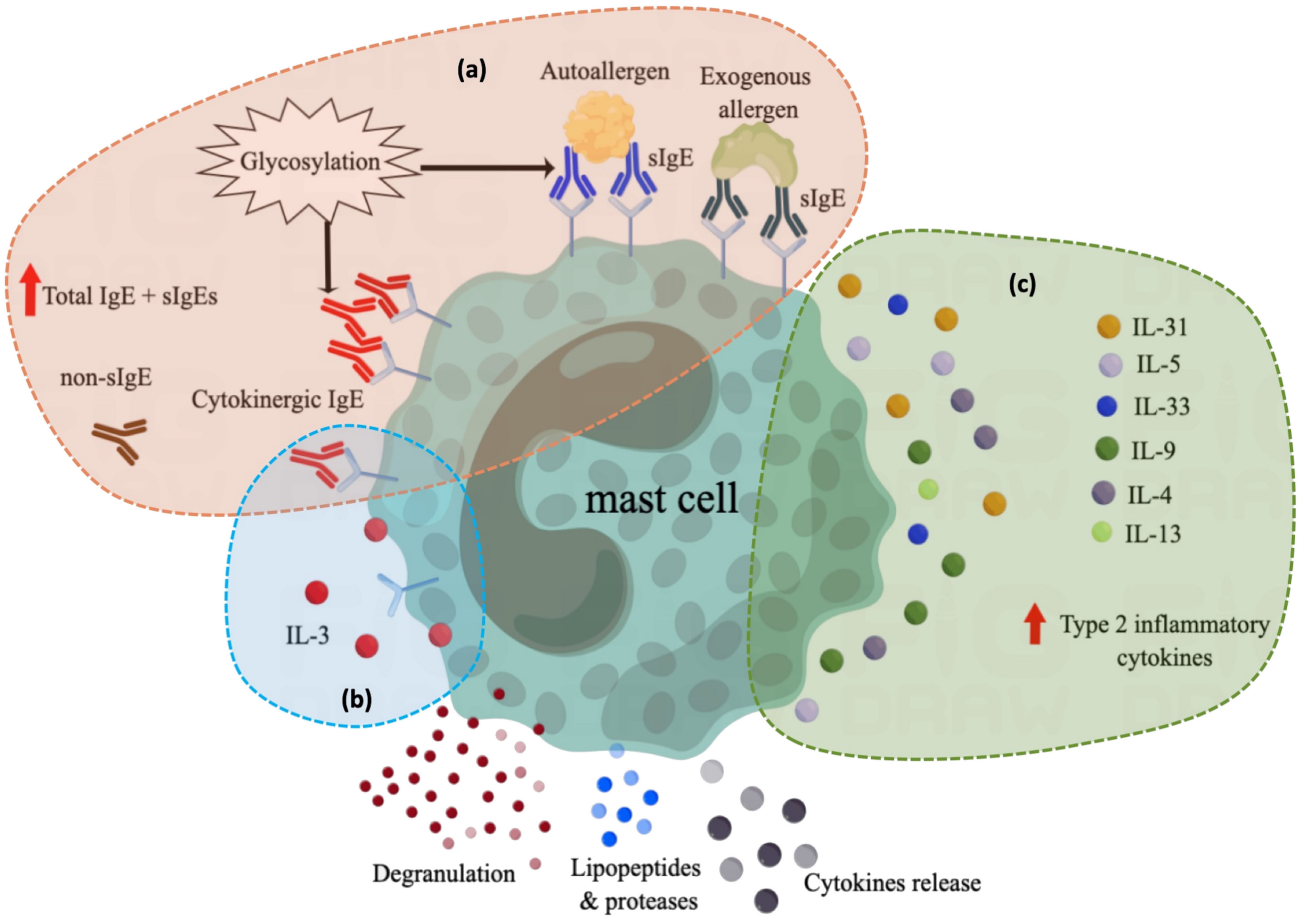
Overview of current understanding of the role of atopy in the pathogenesis of CU. **(A)** In atopic conditions, elevated total IgE levels or the production of various specific IgE induce mast cell activation through different mechanisms, including exogenous allergen-specific IgE and autoallergen-specific IgE, which can activate mast cells by binding to allergens. cytokinergic IgE can activate mast cells through self-cross-linking. Both autoallergen-specific IgE and cytokinergic IgE are modified by glycosylation, which affects their lipophilicity and regulates their binding ability. Although the function of non-specific IgE is unknown, their high levels in atopic contexts may also contribute to mast cell activation or instability. Antibody in green: specific IgE against exogenous allergens; Antibody in blue: specific IgE against autoantigens; Antibody in red: cytokinergic IgE; Antibody in brown: non-specific IgE that has not yet been identified as a specific allergen and whose function is not well defined. **(B)** After IgE binding alone, the autocrine IL-3 secreted by mast cells can positively regulate the survival and function of mast cells; **(C)** Mast cell reactivity is increased in the context of type 2 inflammation. sIgE, specific IgE for exogenous and autoallergens; non-sIgE, nonspecific IgE of unknown function.

Several mechanisms of atopy in patients with CU have been proposed (**Figure 1**). First, IL-3 may play a pro-inflammatory role by enhancing the action of IgE (100). Hide et al. reported that basophil activation induced by high concentrations of IgE is enhanced by physiological concentrations of interleukin (IL)-3 (101), and IgE binding alone can positively regulate the expression of FcϵRI on the surface of MCs by inducing autocrine IL-3, promoting MC survival (102). In the second proposed mechanism, highly cytokinergic IgE (HC-IgE) directly activates MCs or basophils independent of allergens (103). Mouse NS1 hybridoma SPE-7 IgE is an anti-dinitrophenyl antibody and is the most potent HC-IgE discovered to date (104). Bax et al. reported that HC-IgE can self-crosslink to form trimers or multimers that activate MCs (103, 104). Thirdly, IgE is highly glycosylated, and its level of glycosylation has an important effect on its lipophilicity (105). IgE with high lipophilicity expresses polyreactivity to various auto-allergens including dsDNA, TG, TF, TPO, and other histamine-releasing factors, inducing MC degranulation (106). HC-IgE is also related to higher lipophilicity, promoting IgE aggregation and stacking and inducing crosslinking with FcεRI without antigen binding (103).

Most previous studies have focused on the activation phenomenon and crosslinking mechanism of IgE, though studies regarding the intracellular molecular mechanism underlying HC-IgE are rare. HC-IgE induces a series of reactions observed upon IgE-mediated allergen stimulation, such as Ca^2+^ mobilization and phosphorylation of PI3K and MAPK, despite some differences in intensity or time course. These findings suggest that the two may share common signaling pathways (96); however, the similarities require further research. Other studies (107, 108) reported inconsistent miRNA and mRNA profiles between sensitized and activated MCs, suggesting that the intracellular signaling pathways downstream of FcϵRI may differ. Therefore, atopy in patients with elevated IgE levels or sensitization to IgE alone may affect the function and activity of MCs or basophils via multiple pathways, which may contribute to the onset and recurrence of CU symptoms.

## 7 Atopic inflammation in CU

Atopic inflammation, mainly type 2 inflammation, is caused by type 2 immune dysregulation, which is characterized by the expansion of Th2 cells and eosinophils and the excessive production of related cytokines, such as IL-4, IL-13, IL-5, IL-9, IL-31, and IL-33 (109). An increasing number of studies have shown that CU is not a simple histamine-mediated disease, but an inflammatory disease (110). In addition to MCs, activation of a variety of inflammatory cells and changes in a large number of inflammatory mediators are involved, and there is increasing evidence that the inflammatory response to CU is biased toward type 2 inflammation (110).

The inflammatory response to CU can be broadly divided into local and systemic responses. Locally, a particularly pronounced type 2 inflammation pattern has been observed at the wheal lesion sites (111, 112). Histological studies have reported that CU wheals are peri-vascularly infiltrated by mononuclear cells and eosinophils (113, 114), which differs from the pathological appearance of wheal-like lesions in the setting of urticarial vasculitis (115). The infiltration of eosinophils in CSU typically manifests as activation, suggesting a molecular immunopathology similar to the late-phase reaction of cutaneous allergies (113). Immunohistochemical studies suggest an increase in the number of IL-5-positive cells, a chemotactic factor for eosinophils, in the skin lesions of patients with CSU (116). Likewise, IL-33-positive cells, such as macrophages, MCs, endothelial cells, and fibroblasts, are also significantly increased in the dermis of CSU skin lesions (117), while IL-33 plays an important role in type 2 immunity and allergic diseases (118).

In systemic inflammation, multiple type 2 inflammatory mediators are significantly increased in the serum and plasma of patients with CU. As a key cytokine in atopy and a marker of type 2 inflammation, IL-4 is significantly increased in the serum of patients with atopy (119). Some studies have detected significantly increased IL-4 levels in patients with CU (120–122), though these studies did not further clarify whether the patients had atopy. In addition, if patients with atopic CU were divided into separate groups, the IL-4 levels may be even higher. IL-13 shares a receptor subunit with IL-4 and is a pathogenic cytokine involved in Th2 inflammation (123). A previous study of our team reported that the plasma levels of IL-13 in patients with CSU were significantly higher than those in healthy controls (124). Caproni et al. and Bae et al. also demonstrated that the levels of IL-13 in the peripheral blood of patients with CU were higher than those in healthy individuals (125, 126). The roles of IL-9 and T helper 9 (Th9) Cells in allergic diseases have received increasing attention, and IL-9 is recognized as an important factor in type 2 inflammation (127). A pilot study reported elevated serum IL-9 levels in patients with CSU (128). In addition to its high expression in local skin lesions, IL-5 is also significantly more abundant in the serum of patients with CU (129). The serum levels of IL-31, which also promotes Th2 inflammation, were higher in patients with CSU than in healthy controls, but lower than those in patients with AD (130, 131). Based on the analysis of local and systemic inflammatory activation, CU effector cells, such as MCs and basophils, have reduced activation thresholds and higher reactivity in the context of atopic inflammation.

## 8 Biologics in atopic diseases and CU

In recent years, the development of new drugs, especially biologics, for the treatment of CU has reflected the importance of atopy in the setting of CU. As an important factor in allergic diseases, IgE is a diagnostic biomarker and a potential therapeutic target for the treatment of atopic diseases (132). Omalizumab, a monoclonal antibody (mAb) targeting free IgE, was initially approved for the treatment of allergic asthma and was found to elicit a good therapeutic response in patients with refractory CSU. Omalizumab has now been approved as a second-line treatment for patients with CSU who are resistant to H1 antihistamines and is an effective treatment for refractory CIndU (133). Ligelizumab, a next-generation humanized monoclonal anti-IgE antibody, has more than 40 times greater affinity for IgE than omalizumab. A phase 2 trial showed that ligelizumab was more effective than omalizumab for the treatment of CSU; however, this advantage was not confirmed in a phase 3 trial (134). Ligelizumab has also been studied in other allergic conditions, such as food allergies and asthma, though it is not more effective than omalizumab for the treatment of severe asthma (135, 136). UUB-221, an IgE neutralizing mAb distinct from omalizumab and llizumab, exhibits CD23-mediated IgE downregulation and relieves urticaria symptoms (137). Biologics targeting type 2 inflammatory mediators for the treatment of CU have also been reported. Dupilumab, an iconic biological agent for type 2 inflammation that targets the IL-4 and IL-13 signaling pathways (138), has been suggested to successfully treat patients with refractory CU who are refractory to omalizumab in case reports and case series (139–141). Two phase 3 trials (LIBERTY-CUPID Studies A and B, NCT03749135) that evaluate the efficacy of dupilumab for the treatment of CSU that was uncontrolled by standard-of-care antihistamines (CUPID Study A) and or by standard-of-care antihistamines and omalizumab (CUPID Study B) have been completed. The partial results suggest that dupilumab elicits clinical improvement in patients with CSU with H1 antihistamine resistance and is well-tolerated. The use of dupilumab for the treatment of CSU in adults and adolescents was recently accepted for Food and Drug Administration review in the United States. In addition, a phase 3 trial was completed to evaluate the use of dupilumab in CholU (NCT03749148); however, the results have not been published. Dupilumab is an effective and safe “off-label” treatment for CholU (142). Other type 2 inflammatory factors may also be potential targets for CU treatment. Humanized anti-IL-5 mAb reslizumab has been reported as effective in a patient with CSU and cold urticaria (143). Ixarelimab, a fully human mAb that simultaneously inhibits IL-31 and oncostatin M, is being trialed in pruritic diseases including CSU (NCT03858634). Overall, based on the overlap between CU and atopic diseases and the role of atopy in the pathogenesis of CU, an increasing number of biologics targeting atopic diseases will be developed for the treatment of CU.

## 9 Conclusion

As CU is not considered an atopic disease, the relationship between atopy and CU is often overlooked. However, the prevalence of atopy in patients with CU is high and correlates with the clinical characteristics of the disease. An increasing number of studies have found more specific IgEs against various auto-allergens and some exogenous allergens in patients with CU. The clinical value of total IgE in patients with CU is being further explored. Therefore, IgE is considered a critical and promising therapeutic target for CU and other atopic diseases (144). Additionally, an increasing number of studies have shown that CU is associated with a certain degree of type 2 inflammatory activation, whether local or systemic, which is a common phenomenon in other atopic diseases. The effectiveness of targeted therapies for type 2 inflammation in the treatment of CU is gradually increasing. Therefore, by comprehensively summarizing the existing research progress, the role of atopy in the pathogenesis of CU can be estimated (**Figure 1**). In summary, although the available evidence is insufficient to support atopy as a direct trigger for CU, the presence of atopy increases the risk of CU and induces MCs to become more active, aggravating CU symptoms.

Based on the available evidence and progress, several clinical statements can be made. In contrast to aiCSU, clinical subtypes of CU, such as atopic CSU or atopic CIndU, may be recognized; however, more clinical studies are needed to clarify the characteristics of these subtypes. In addition, cross-antigens between exogenous allergens and CU auto-allergens or a correlation between exogenous allergens and CU auto-allergens may exist. In the future, the presence of auto-allergens may be confirmed by testing for common exogenous allergens, and specific immunotherapy of common allergens may alleviate the symptoms of atopic CU caused by specific IgEs of auto-allergens. An understanding of the effect of IgE sensitization or type 2 inflammation on the pathogenesis of urticaria, such as in MCs and basophils, may allow for the discovery of therapies (such as omalizumab, allergen-specific immunotherapy, dupilumab, and Jak1 inhibitors) that target IgE sensitization and type 2 inflammatory factors in the setting of CU.

## Author contributions

QC: Conceptualization, Investigation, Writing – original draft. XY: Data curation, Investigation, Writing – original draft. BN: Conceptualization, Supervision, Validation, Writing – review & editing. ZS: Conceptualization, Funding acquisition, Supervision, Writing – review & editing.
